# Vascular mechanotransduction data in a rodent model of diabetes: Pressure-induced regulation of SHP2 and associated signaling in the rat inferior vena cava

**DOI:** 10.1016/j.dib.2017.09.028

**Published:** 2017-09-20

**Authors:** Kevin M. Rice, Nandini D.P.K. Manne, Ravikumar Arvapalli, Gautam K. Ginjupalli, Eric R. Blough

**Affiliations:** a Center for Diagnostic Nanosystems, Marshall University, Huntington, WV, USA; b Department of Internal Medicine, Joan C. Edwards School of Medicine, Marshall University, Huntington, WV, USA; c Biotechnology Graduate Program West Virginia State University, Institute, WV, USA; d Department of Health and Human Service, School of Kinesiology, Marshall University, Huntington, WV, USA; e Department of Public Heath, Marshall University, Huntington, WV, USA; f Department of Pharmaceutical Sciences and Research, School of Pharmacy, Marshall University, Huntington, WV, USA; g Department of Pharmacology, Physiology and Toxicology, Joan C. Edwards School of Medicine, Marshall University, Huntington, WV, USA

## Abstract

The effect of diabetes on vascular mechano-transductive response is of great concern. Given the higher rate of vein graft failures associated with diabetes, understanding the multiple cellular and molecular events associated with vascular remodeling is of vital importance. This article represents data related to a study published in Cardiovascular Diabetology [1] (Rice et al., 2006) and Open Journal of Endocrine and Metabolic Diseases [2] (Rice et al., 2015) evaluating the effect of pressurization on rat inferior venae cavae (IVC). Provided within this articles is information related to the method and processing of raw data related to our prior publish work and Data in Brief articles [3], [4] (Rice et al., 2017), as well as the evaluation of alternation in SHP-2 signaling and associated proteins in response to mechanical force. IVC from lean and obese animals were exposed to a 30 min perfusion of 120 mm Hg pressure and evaluated for changes in expression of SHP2, BCL-3, BCL-XL, HSP 27, HSP 70, and PI3K p85, along with the phosphorylation of SHP-2 (Tyr 542).

**Specifications Table**

**Table:** 

Subject area	*Biology*
More specific subject area	*Cardiovascular diabetic surgical tissue response*
Type of data	*graph, figure*
How data was acquired	*immunoblotting*
Data format	*analyzed*
Experimental factors	*IVC mounted vessels were subjected to 120 mm Hg of pressure for 30 minutes. Protein was then isolated from tissue for western blot analysis.*
Experimental features	*IVC obtained from Lean and Obese male Zucker rats were used in this experiment*
Data source location	*The data is presented within this document*
Data accessibility	*Data is wit this article and is related to articles published and in review*[1], [2], [3], [4]

**Value of the data**

• The data presented in this Brief is vital to understanding the effect of diabetes on tissue
• This data gives insight into the how diabetes alters tissue response to stimuli
• The data can provide comprehensive analysis of the effect of diabetes on vascular signaling in vein transplant surgery
• These data provides a more thorough understanding of the SHP-2 involvement in pressure mediated signaling in both diabetic and lean IVC

## 1 Data

### 1.1 SHP-2

**Fig. 1:**
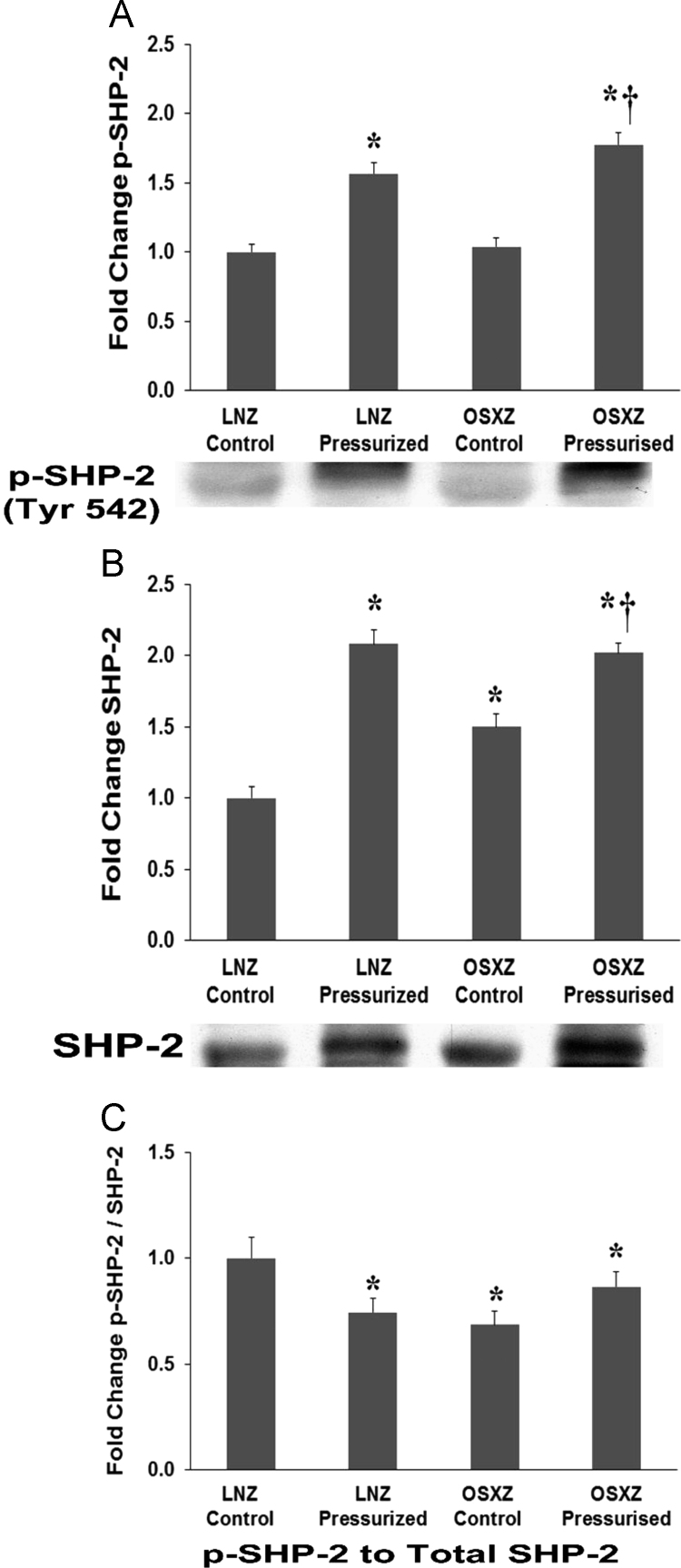
Diabetes alters loading-induced SHP2 expression and phosphorylation rat inferior vena cava. The basal (control) and pressure-induced expression and phosphorylation of SHP2 in venae cavae from non-diabetic lean Zucker (LNZ) and diabetic obese syndrome X Zucker (OSXZ) rats. * Significantly different from unloaded venae cavae within the same group (*p* < 0.05). † Significantly different from corresponding LNZ venae cavae (*p* < 0.05). *n* = 6/group.

To determine the effect of pressurization of inferior vena cava (IVC) from diabetic male obese syndrome-X Zucker (OSXZ) diabetic and nondiabetic male normal lean Zucker (LNZ) animals we evaluated the expression of Tyrosine-protein phosphatase non-receptor type 11 (PTPN11) also known as Src homology region 2 domain-containing phosphatase-2 (SHP-2) [5]. IVCs obtained from the OSXZ control group showed significant increase in the basal expression of SHP-2 when compared to the LNZ control animals (50 ± 9.3% *p*<0.05). Pressurization resulted in a significant increase in SHP-2 in the LNZ IVC (108 ± 10.2% *p*<0.05) and OSXZ IVC (52 ± 6.6% *p*<0.05) (Fig. 1-B). However the pressure induced elevation in the OSXZ IVC was significantly less than that of the lean (56 ± 16.8% *p*<0.05) (Fig. 1-B). Compared to LNZ controls SJP-2 basal phosphorylation at tyrosine 542 demonstrated no significant difference in the OSXZ IVC. Pressurization of the IVC resulted in a significant increase in the phosphorylation of SHP-2 in the LNZ IVC (57 ± 8.1% *p*<0.05) and the OSXZ IVC (74 ± 8.9% *p*<0.05) (Fig. 1-A). The ratio of p-SHP-2 toSHP-2 demonstrated a significant decrease in the basal levels of p-SHP-2 to SHP-2 in the OSXZ IVC (31 ± 6.2% *p*<0.05) compared to LNZ controls. Pressurization decrease the LNZ (26 ± 6.6% *p*<0.05) ratio of p-SHP-2 to SHP-2 but failed to change the OSXZ ratio of p-SHP-2 to SHP-2, this absence of a pressure induced decrease in the OSXZ IVC was significantly different compared to lean (Fig. 1-C).

### 1.2 BCL-3

**Fig. 2:**
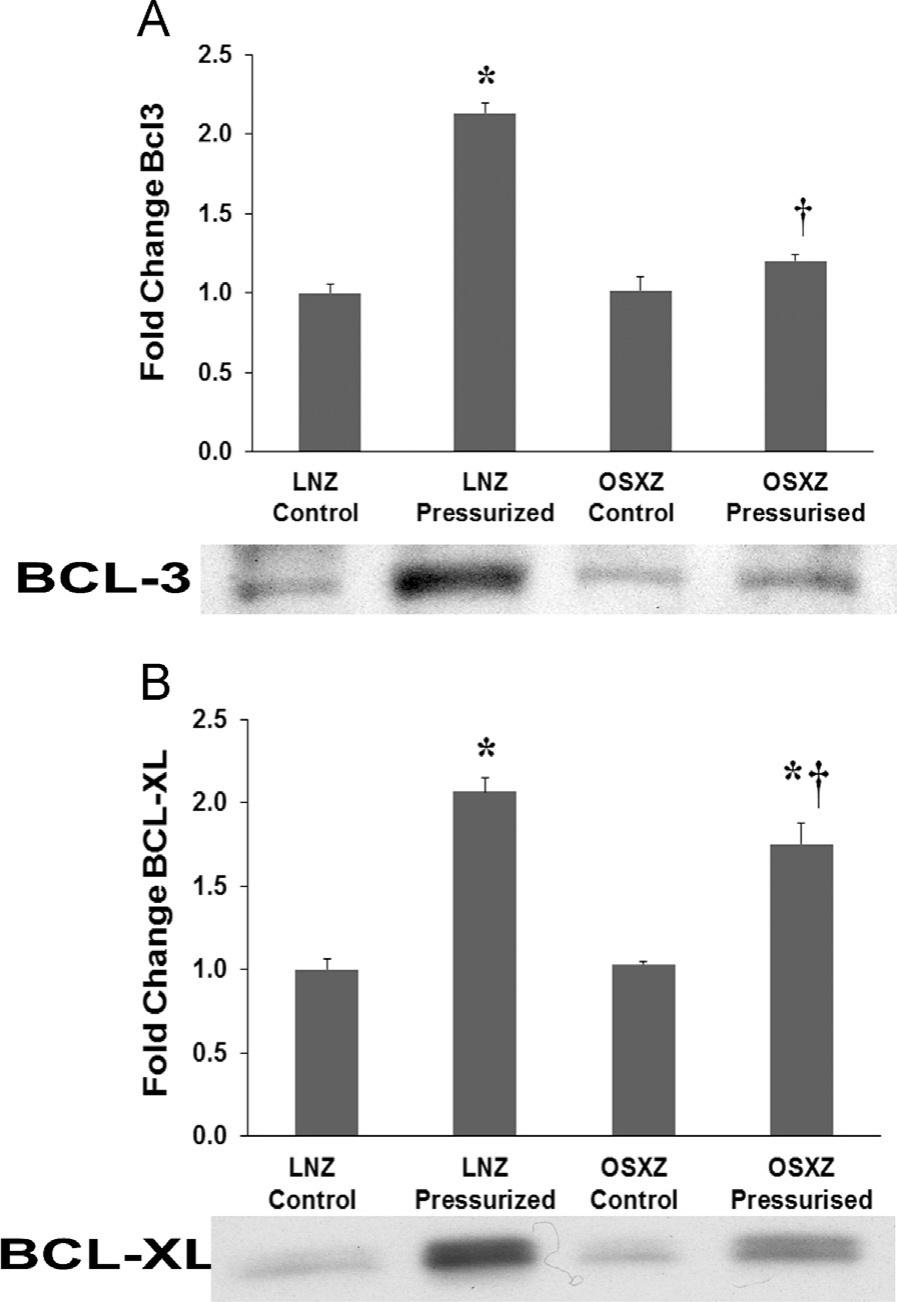
*Diabetes alters loading-induced BCL-3 and BCL-XL expression in rat inferior vena cava.* The basal (control) and pressure-induced expression of BCL-3 and BCL-XL in venae cavae from non-diabetic lean Zucker (LNZ) and diabetic obese syndrome X Zucker (OSXZ) rats. * Significantly different from unloaded venae cavae within the same group (*p*< 0.05). † Significantly different from corresponding LNZ venae cavae (*p*< 0.05). *n* = 6/group.

BCL-3 is a member of the IκB family of NFκB and is regulated by GSK3 [6]. SHP-2 is activated by GSK3 inhibition but is not a direct target of GSK3 [7]. To investigate the possible link between SHP-2 and BCL-3 through GSK3 we looked at changes in BCL-3 expression. OSXZ control IVC showed no significant change in BCL-3 basal expression when compared to LNZ control. Examination of the effect of pressurization of inferior vena cava expression of BCL-3 demonstrated significant increase in LNZ (113±6.4% *p*<0.05) but no change in OSXZ animals, this absence of a pressure induced increase in the OSXZ IVC was significantly different compared to lean (Fig. 2-A).

### 1.3 BCL-XL

The BCL-2 homolog BCL-XL prominent pro-survival functions by binding to its multi-domain pro-apoptotic counterparts BAX and BAK, thus preventing the formation of lethal pores in the mitochondrial outer membrane [8]. The vasoactive peptide angiotensin II (Ang II) activated SHP-2 inhibits nucleolin binding to BCL-XL mRNA thereby altering the equilibrium and expression of anti- and pro-apoptotic regulators [9]. OSXZ control IVC showed no significant change in BCL-XL basal expression when compared to LNZ control. Examination of the effect of pressurization of IVC expression of BCL-XL demonstrated significant increase in LNZ (106 ± 8.3% *p*<0.05) and OSXZ (72 ± 12.7% *p*<0.05). However the pressure induced elevation in the OSXZ IVC was significantly less than that of the lean (34 ± 21.0% *p*<0.05) (Fig. 2-B).

### 1.4 HSP 27

**Fig. 3:**
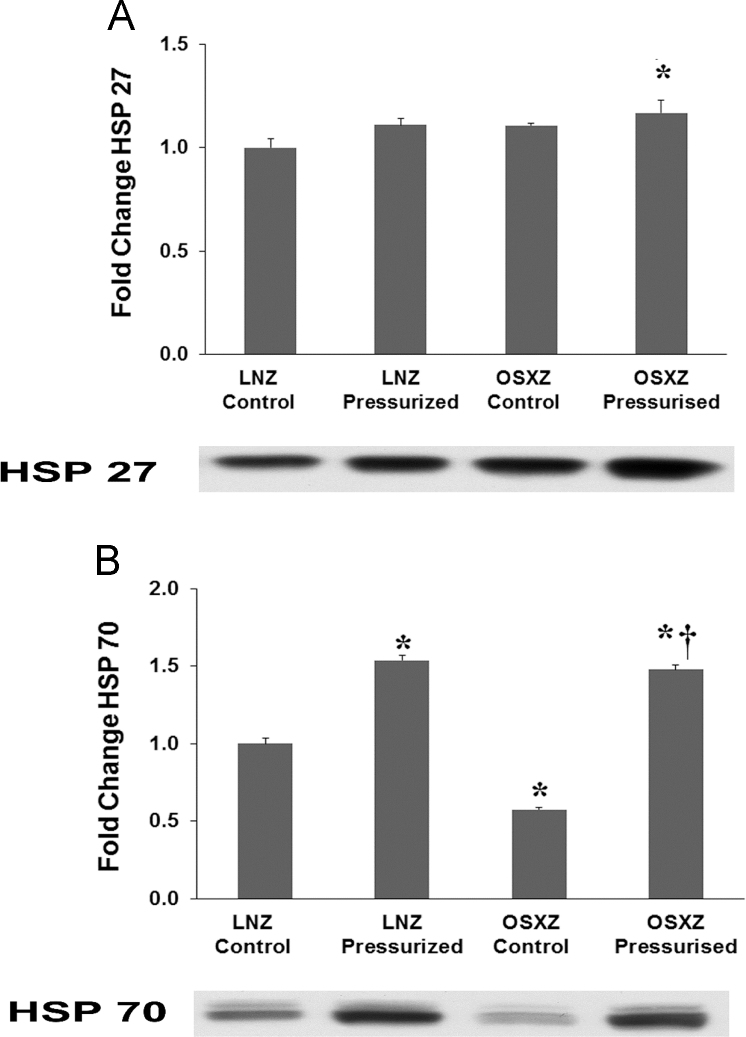
*Diabetes alters loading-induced HSP 70 expression but not HSP 27 expression in rat inferior vena cava.* The basal (control) and pressure-induced expression of HSP 27 and HSP 70 in venae cavae from non-diabetic lean Zucker (LNZ) and diabetic obese syndrome X Zucker (OSXZ) rats. * Significantly different from unloaded venae cavae within the same group (*p*< 0.05). † Significantly different from corresponding LNZ venae cavae (*p*< 0.05). *n* = 6/group.

The small heat shock protein HSP 27 has been shown to be involved in a number of cellular processes. Recently research has indicated that it is required for proper heart tube formation [10]. SHP-2 inhibition has been shown to have no effect on the expression of HSP 27 a known marker for cardiac differentiation [11]. To confirm that HSP 27 and SHP-2 demonstrated no association in vascular remodeling we looked at the expression of HSP 27 in the pressurized IVC. OSXZ control IVC showed no significant change in HSP 27 basal expression when compared to LNZ control. Examination of the effect of pressurization of IVC expression of SHP 27 demonstrated no significant increase in LNZ or OSXZ (Fig. 3-A).

### 1.5 HCP 70

The 70 kDa heat shock proteins (HSP 70) is a ubiquitously expressed chaperone protein activated by various cellular stresses including oxygen-free radicals, inflammation, heat shock, oncogenes, and proto-oncogenes [12]. HSP70 play an important role in protecting the cells from stress and regulating the cell's machinery for protein folding [13], [14]. HSP70 has been shown to bind SHP-2 and effect SHP-2 related signaling [12], [15]. IVCs obtained from the OSXZ control group showed a significant decrease in the basal expression of HSP 70 when compared to the LNZ control animals (43 ± 1.4% *p*<0.05). Pressurization resulted in a significant increase in HSP 70 in the LNZ IVC (54 ± 2.9% *p*<0.05) and OSXZ IVC (91 ± 3.0% *p*<0.05) (Fig. 1-B). However the pressure induced elevation in the OSXZ IVC was significantly greater than that of the lean (37 ± 6.9% *p*<0.05) (Fig. 3-B).

### 1.6 PI3K p85

**Fig. 4:**
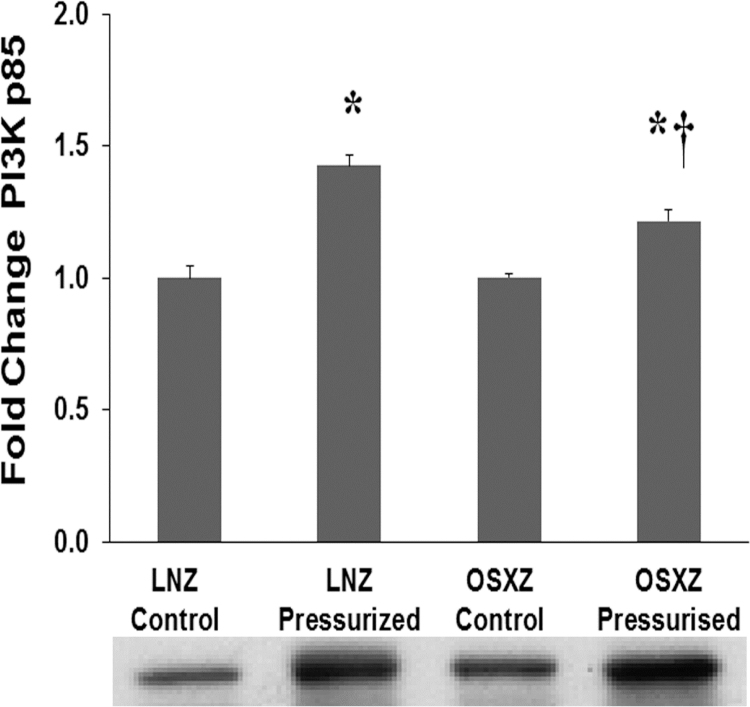
*Diabetes alters loading-induced PI3K p85 expression in rat inferior vena cava.* The basal (control) and pressure-induced expression of PI3K p85 in venae cavae from non-diabetic lean Zucker (LNZ) and diabetic obese syndrome X Zucker (OSXZ) rats. * Significantly different from unloaded venae cavae within the same group (*p*< 0.05). † Significantly different from corresponding LNZ venae cavae (*p*< 0.05). *n* = 6/group.

Phosphatidylinositol-4,5-bisphosphate 3-kinase (PI3K) is a family of enzymes involved in proliferation, motility, cell growth, differentiation and intracellular trafficking. PI3K is composed of a heterodimer between a p85 regulatory subunit and a p110 catalytic subunit [16]. Data has shown that phosphorylated SHP-2 can associate with the p85 regulatory subunit of PI3K (PI3K p85) and enhance PI3K activity [17]. OSXZ control IVC showed no significant change in PI3K p85 basal expression when compared to LNZ control. Pressurization resulted in a significant increase in PI3K p85 in the LNZ IVC (43 ± 4.1% *p*<0.05) and OSXZ IVC (21 ± 4.5% *p*<0.05). However the pressure induced elevation in the OSXZ IVC was significantly less than that of the lean (21 ± 8.6% *p*<0.05) (Fig. 4).

## 2 Experimental design, materials and methods

### 2.1 Animals

Young (10 week, *n*=12) male normal lean Zucker (non-diabetic) (LNZ) and young (10 week, *n*=12) male obese syndrome-X Zucker (diabetic) (OSXZ) rats were obtained from the Charles River Laboratories and barrier housed one per cage in an AAALAC approved vivarium. All procedures were conducted in strict accordance with the Public Health Service policy on animal welfare. Housing conditions consisted of a 12 H: 12 H dark-light cycle and temperature was maintained at 22±2 °C. Animals were provided food and water *ad libitum*. Rats were allowed to recover from shipment stress for at least two weeks before the commencement of experimentation during which time the animals were carefully observed and weighed weekly. All procedures were performed in accordance with the Guide for the Care and Use of Laboratory Animals as approved by the Council of the American Physiological Society and the Animal Use Review Board of Marshall University. [1], [2], [3], [4].

### 2.2 Materials

Antibodies against Pi3K p85 [cat # 4257], HSP 27 [cat #2442], HSP 70 [cat #4872], SHP-2 [cat #3752], p-SHP-2 (Tyr 542) [cat #3751], and Bcl-XL [cat #2762], p70 S6 Kinase Control Cell Extracts [cat #9203], mouse IgG and rabbit IgG were purchased from Cell Signaling Technology (Beverly, MA). Purchased antibodies against Bcl-3 (H-146) [sc-13038], from Santa Cruz Biotechnology (Santa Cruz, CA). SDS-PAGE precast gels were purchased from Lonza (Rockland, ME). Enhanced chemiluminescence (ECL) western blotting detection reagent was purchased from Amersham Biosciences (Piscataway, NJ). All other chemicals were from Sigma (St. Louis, MO).

### 2.3 Inferior vena cava preparation

Inferior venae cavae and experimental design were performed as described by Rice et. al [1], [2], [3], [4].

### 2.4 Immunoblot analysis

Immunoblotting was performed according to the protocol outlined by Rice et al. [1], [2], [3], [4].

## 3 Data analysis

Data were analyzed using the Sigma Stat 3.0 and results were presented as mean ± SEM. A one-way analysis of variance (ANOVA) was performed for overall comparisons followed by the Student-Newman-Keuls post hoc test to determine differences between groups. The level of significance accepted *a priori* was ≤ 0.05.
